# Eye drop delivery of pigment epithelium-derived factor-34 promotes retinal ganglion cell neuroprotection and axon regeneration

**DOI:** 10.1016/j.mcn.2015.08.001

**Published:** 2015-09

**Authors:** Vasanthy Vigneswara, Maryam Esmaeili, Louise Deer, Martin Berry, Ann Logan, Zubair Ahmed

**Affiliations:** Neurotrauma Research Group, Neurobiology Section, School of Clinical and Experimental Medicine, College of Medical and Dental Sciences, University of Birmingham, Birmingham, B15 2TT, UK

**Keywords:** PEDF eye drops, Retinal ganglion cells, Neuroprotection, Axon regeneration, Neurite outgrowth

## Abstract

Axotomised retinal ganglion cells (RGCs) die rapidly by apoptosis and fail to regenerate because of the limited availability of neurotrophic factors and a lack of axogenic stimuli. However, we have recently showed that pigment epithelium-derived factor (PEDF) promotes RGC survival and axon regeneration after optic nerve crush injury. PEDF has multiple fragments of the native peptide that are neuroprotective, anti-angiogenic and anti-inflammatory. Here we investigated the neuroprotective and axogenic properties of a fragment of PEDF, PEDF-34, in retinal neurons *in vitro* and when delivered by intravitreal injection and eye drops *in vivo*. We found that PEDF-34 was 43% more neuroprotective and 52% more neuritogenic than PEDF-44 *in vitro*. Moreover, *in vivo*, intravitreal delivery of 1.88 nM PEDF-34 was 71% RGC neuroprotective at 21 days after optic nerve crush compared to intact controls, whilst daily eye drops containing 1.88 nM PEDF-34 promoted 87% RGC survival. After topical eye drop delivery, PEDF-34 was detected in the vitreous body within 30 min and attained physiologically relevant concentrations in the retina by 4 h peaking at 1.4 ± 0.05 nM by 14 days. In eye drop- compared to intravitreal-treated PEDF-34 animals, 55% more RGC axons regenerated 250 μm beyond the optic nerve lesion. We conclude that daily topical eye drop application of PEDF-34 is superior to weekly intravitreal injections in promoting RGC survival and axon regeneration through both direct effects on retinal neurons and indirect effects on other retinal cells.

## Highlights

• PEDF-34 is more neuroprotective and neuritogenic than PEDF-44.
• PEDF-34 is more neuroprotective and neuritogenic than full-length PEDF.
• PEDF-34 can reach the retina after topical application to the eyes.
• PEDF-34 eye drops are more neuroprotective and axogenic than intravitreal injection.

## 1 Introduction

The 50 kDa neurotrophic factor pigment epithelium-derived factor (PEDF) is a member of the serpin superfamily, first isolated from foetal retinal pigment epithelial cells but expression in normal choroid, ciliary body, corneal epithelium, retinal ganglion cells (RGCs) and photoreceptors has since been reported (Barnstable and Tombran-Tink, 2004; Karakousis et al., 2001; Lange et al., 2008; Ogata et al., 2002; Pang et al., 2007; Tombran-Tink and Barnstable, 2003; Unterlauft et al., 2012). Other tissues/cells including the brain, spinal cord, skeletal muscle, heart, endothelial cells and osteoblasts also express PEDF (Akiyama et al., 2010; Longeras et al., 2012; Rychli et al., 2010; Sanagi et al., 2010; Tombran-Tink and Barnstable, 2003). In addition to putative anti-angiogenic, anti-inflammatory, anti-oxidative and anti-tumorigenic properties, PEDF is also neuroprotective for CNS neurons (Cao et al., 1999; Dawson et al., 1999; Duh et al., 2002; Feng et al., 2013; Ho et al., 2011; Zhang et al., 2006; Zhu and Zou, 2012). For example, in many retinal pathologies, PEDF is secreted by reactive Müller cells and astrocytes and is neuroprotective for RGC and photoreceptors (Cao et al., 2001; Lange et al., 2008; Li et al., 2006; Zhu and Zou, 2012).

Axotomised RGCs are not viable unless treated with neurotrophic factors such as brain-derived neurotrophic factor, ciliary neurotrophic factor and glial cell line-derived factor (Ahmed et al., 2006b; Benowitz and Yin, 2007; Watanabe et al., 2003; Yip and So, 2000). Recently, we confirmed that, after optic nerve crush (ONC), PEDF is RGC neuroprotective and axogenic (Vigneswara et al., 2013), whilst other neuroprotective and anti-angiogenic N-terminal fragments of the native peptide exist (Bilak et al., 2002; Gvritishvili et al., 2010; Li et al., 2006; Mirochnik et al., 2009) including the neuroprotective amino acid residues 78–121, anti-angiogenic residues 16–26, 24–57, 44–77, 60–77 and 78–94 (Alberdi et al., 1999; Bilak et al., 2002; Filleur et al., 2005; Liu et al., 2012) and residues 58–101 which promote the differentiation of PC3 cells into neuro-secretory neurons (Filleur et al., 2005). Residues 78–101 constitute the PEDF-44 fragment, which is neuroprotective for embryonic rat spinal motor neurons, whilst the PEDF-34 fragment, comprising residues 44–77 is not neuroprotective (Bilak et al., 2002). In this study, we show that PEDF-34 is a more potent retinal neuron/RGC neuroprotective/neuritogenic fragment than equimolar concentrations of PEDF-44 *in vitro*. PEDF-34 was also more neuroprotective and axogenic *in vivo*, after topical eye drop application compared to intravitreal injection.

## 2 Materials and methods

### 2.1 Experimental design: *in vitro* experiments

For all *in vitro* experiments, adult 6–8-week-old female Sprague–Dawley rats weighing 170–220 g were used. Culture experiments included retinal cells treated with: (1), Neurobasal-A (NBA) alone (Control); and equimolar concentrations of (2), 2.16; (3) 5.4; (4), 10.8 and (5), 21.6 pM PEDF-34/-44; and (6), ciliary neurotrophic factor (CNTF: Peprotech, London, UK), as a positive control at an optimal neuroprotective/axogenic concentration of 440.5 pM, established by us previously (Ahmed et al., 2009; Douglas et al., 2009). All treatments were added to cultures on the following day (16–18 h after cell seeding) when cells had adhered to the culture vessel. All experiments were performed in triplicate and repeated on three independent occasions.

### 2.2 Experimental design: *in vivo* experiments

In the first experiment, groups of 6 rats/treatment (*i.e.* 12 eyes/treatment) were used to determine RGC survival by FluroGold (FG) backfilling after weekly intravitreal PEDF-34 injections, comprising: (1), intact; (2), ONC + vehicle (phosphate buffered saline (PBS); 0 nM PEDF-34); (3), 0.268 nM PEDF-34; (4), 0.67 nM PEDF-34; (5), 1.34 nM PEDF-34; (6), 1.88 nM PEDF-34 and (7) 2.68 nM PEDF-34. These equimolar concentrations were chosen since they are comparable to those used in our previous study with full length PEDF (Vigneswara et al., 2013). In a separate set of experiments comprising the same treatment groups, 6 rats/treatment (*i.e.* 12 eyes/optic nerves/treatment) were used to evaluate RGC axon regeneration by immunostaining with growth-associated protein-43 (GAP-43) in the optic nerve.

To test the accumulation of PEDF-34 in the eye by western blot, 6 rats (12 eyes)/time point received pre-optimised 1.88 nM PEDF-34 by a single eye drop onto the anterior surface of the eye and at 0.5, 4 and 24 h after treatment, animals were killed by rising CO_2_ levels and retinae were harvested and analysed by western blot analysis. To confirm the accumulation of PEDF-34 in the eye by enzyme-linked immunosorbent assay (ELISA), 6 animals (12 eyes)/time point/treatment were used to analyse PEDF-34 levels in the vitreous and retina over the first 24 h. To test the accumulation of PEDF-34 in the vitreous and retina after daily eye drop delivery, 6 animals (12 eyes)/day were used to analyse the concentration of PEDF-34 by ELISA for a period of 28 days.

In further experiments, 12 rats (24 eyes/optic nerves)/treatment received 1.88 nM PEDF-34 eye drops daily. Six rats (12 eyes)/treatment were used to assess RGC survival at 21 days using FG backfilling, whilst the remaining 6 rats (12 eyes/optic nerves)/treatment were used to evaluate RGC axon regeneration at 21 days by GAP-43 immunohistochemistry.

### 2.3 PEDF-34 synthesis

In initial experiments, PEDF-34 was purchased from Phoenix Europe GmbH, Karlsruhe, Germany but the majority of *in vitro* and *in vivo* experiments were performed with in-house synthesised PEDF-34. The PEDF-34 peptide spanning from amino acids 44 to 77 of the N-terminus of the PEDF protein was chemically synthesised in a solid phase peptide synthesiser, purified by HPLC and characterised by mass spectrometry (Longeras et al., 2012). The similar activity of commercially purchased compared to in-house synthesised PEDF-34 was confirmed in retinal cultures.

### 2.4 Adult retinal cultures

Mixed adult rat retinal cultures containing enriched populations of retinal neurons, including RGCs, were prepared from 6–8-week-old adult female Sprague–Dawley rats, as described previously (Ahmed et al., 2006b, 2009, 2010; Douglas et al., 2009; Vigneswara et al., 2013). Briefly, retinal cells were dissociated using a Papain dissociation kit according to the manufacturer's instructions (Worthington Biochemicals, Lakewood, NJ, USA). Retinal cells were plated at a density of 125 × 10^3^/well in poly-d-lysine and laminin pre-coated 8-well chamber slides and cultured in NBA supplemented with B27 supplement (all from Invitrogen, Paisley, UK), with appropriate treatments for 4 days at 37 °C and 5% CO_2_ before fixation in 4% paraformaldehyde diluted in PBS for immunocytochemistry, as described previously (Vigneswara et al., 2013, 2014).

### 2.5 Immunocytochemistry of retinal cultures

Fixed cells were washed in several changes of PBS before permeabilisation and blocking of non-specific antibody sites with PBS containing 3% bovine serum albumin (BSA) and 0.1% Triton X-100 (both from Sigma, Poole, UK). Cells were then incubated with monoclonal anti-βIII-tubulin antibody (1:200 dilution; Sigma) for 1 h at room temperature (RT) to detect retinal neurons and their neurites. Cells were then washed in several changes of PBS and incubated with Alexa 488 anti-mouse IgG (1:400 dilution; Invitrogen) for 1 h at RT. After washing in several changes of PBS and mounting in Vectamount containing DAPI (Vector Laboratories, Peterborough, UK), cells were then viewed using a Zeiss epi-fluorescent microscope equipped with an AxioCam HRc and Axiovision Image capture software (all from Zeiss, Hertfordshire, UK). Immunocytochemistry included controls with primary antibody omitted, to set background levels of nonspecific staining (not shown) before image capture.

### 2.6 Retinal neuron survival and neurite outgrowth

The mean number of βIII-tubulin^+^ neurons surviving, with neurites and the mean neurite length were quantified as described by us previously (Vigneswara et al., 2013, 2014). Briefly, chamber slides were anonymised by a second investigator and βIII-tubulin^+^ neuronal soma and neurites quantified in 9 quadrants/well using Axiovision software (Version 4.8; Zeiss) and ImagePro (Version 6.3; Media Cybernetics, Bethesda, MD, USA). Of the 125,000 cells/well plated, the total numbers of neurons in each experiment were determined by βIII-tubulin^+^ immunocytochemistry after allowing 30 min for cells to adhere to glass culture slides. This determines the number of neurons plated/well and is used in each experiment to work out the proportion of surviving βIII-tubulin^+^ neurons. Neurite outgrowth from at least 180 neurons/condition from the 9 wells was measured, except in control (NBA) cultures, in which a total of 108 neurons were measured.

### 2.7 Optic nerve crush (ONC)

All animal procedures were licenced and approved by the United Kingdom Home Office and the University of Birmingham ethical review committee and conformed to the Federation of European Laboratory Animal Associations (FELASA) guidelines. Optic nerves were exposed surgically in anaesthetised adult female Sprague–Dawley rats (170–220 g) through a supraorbital approach and crushed bilaterally using calibrated watchmakers forceps, 2 mm from the lamina cribrosa as described by us previously (Vigneswara et al., 2013, 2014).

### 2.8 Intravitreal injections

Either PBS, or 0.268, 0.67, 1.34, 1.88, or 2.68 nM PEDF dissolved in 5 μl of sterile PBS was injected intravitreally immediately after ONC (0 days) and repeated at 7 and 14 days after ONC with the same doses of PEDF. None of the animals developed cataracts confirming that the lens had not been injured. Animals survived for 19 days before FG back-labelling and killing by rising CO_2_ levels 2 days later, and eyes/optic nerves harvested for retinal FG wholemounts. Further animals were also killed at 21 days after ONC and intravitreal injections of PEDF-34 and cryosectioned for immunohistochemistry.

### 2.9 Eye drops

Eye drops were formulated in 0.9% sterile saline as described previously and the same molar concentrations delivered as for PEDF-34 used for weekly intravitreal injections (Vigneswara et al., 2013). Animals were lightly anaesthetised to prevent blinking and 5 μl of PEDF-34 with the pre-optimised 1.88 nM PEDF-34 was dropped onto the corneal/conjunctival surface daily for up to 28 days. To assess RGC survival and axon regeneration, FG back-labelling was performed at 19 days and animals were killed 2 days later by rising CO_2_ after which eyes were harvested for retinal wholemounts. Further animals were killed at 21 days after ONC and intravitreal PEDF-34 injections, killed by rising CO_2_ levels and prepared for immunohistochemistry, western blot and ELISA.

### 2.10 Retinal wholemounts

Nineteen days after ONC, FG (Cambridge Biosciences, Cambridge, UK) was injected into the optic nerve, between the lamina cribrosa and the ONC site (Vigneswara et al., 2013, 2014). Animals were killed 2 days later and the dissected retinae were immersion-fixed in 4% formaldehyde (TAAB Laboratories, Aldermaston, UK), flattened onto Superfrost Plus microscope slides (VWR International, Lutterworth, UK) by making four equidistal radial cuts to obtain four quadrants, air dried and mounted in Vectamount (Vector Laboratories). The identity of individual retinae was anonymised by a second investigator before image capture using a Zeiss epifluorescent microscope and the number of FG^+^ RGCs counted in ImagePro Version 6.0 (Media Cybernetics) from captured images of 12 rectangular areas, 3 from each quadrant, placed at radial distances from the centre of the optic disc of the inner (1/6 eccentricity), midperiphery (1/2 eccentricity) and outer retina (5/6 eccentricity). The mean densities of FG^+^ RGCs/mm^2^ for each retina were determined.

### 2.11 Tissue preparation and sectioning for immunohistochemistry

Animals were killed with an overdose of CO_2_ and intracardially perfused with 4% formaldehyde, eyes and optic nerves were immersion fixed in 4% formaldehyde for a further 2 h before cryoprotection in a graded series of sucrose. Eyes and optic nerves were embedded in optimal cutting temperature (OCT) compound (Raymond A. Lamb, Eastbourne, UK) and stored at − 80 °C. Subsequently, 15 μm-thick parasagittal eye or longitudinal optic nerve sections were cut using a cryostat (Brights Instruments, Huntingdon, UK), adhered onto glass slides and stored at − 20 °C until required.

### 2.12 Immunohistochemistry

Immunohistochemistry was performed as described by us previously (Vigneswara et al., 2013, 2014). Briefly, sections were thawed, washed at RT in PBS and permeabilised. Non-specific binding sites were blocked in 3% bovine serum albumin (BSA) containing 0.1% Triton X-100 and then sections were incubated in primary antibody diluted in PBS containing 3% BSA and 0.05% Tween-20, overnight at 4 °C (16–18 h). Monoclonal anti-GAP-43 antibody (1:500 dilution; Invitrogen) stained regenerating axons in optic nerve sections (Table 1). Immunohistochemistry negative controls, in which the primary antibody was omitted were included in each run and showed an absence of staining (not shown). Sections were then washed in PBS and incubated with either Alexa Fluor 488 or Texas Red-labelled secondary antibodies (1:400 dilution; Invitrogen) for 1 h at RT, washed in PBS, mounted in Vectamount containing DAPI (Vector Laboratories) and examined with a Zeiss Axioplan 2 fluorescent microscope, equipped with an AxioCam HRc camera and Axiovision software. The negative control sections were used to set the background threshold levels of fluorescence before image capture.

### 2.13 Quantification of axon regeneration

Regenerating axons in the optic nerve were quantified using previously published methods (Leon et al., 2000; Lorber et al., 2009; Vigneswara et al., 2013, 2014). We did not use Cholera toxin B labelling since GAP43 immunohistochemistry to detect regenerating axons is the gold standard method in the rat. In addition, we have recently shown that anterograde tracing with Rhodamine B in the rat is correlated with the number of GAP43^+^ axons detected in both proximal and distal areas after optic nerve crush, suggesting that both methods detect the same regenerated axons (Vigneswara et al., 2014). Briefly, an observer blinded to the identity of the slides counted the number of axons extending beyond a vertical line at 250, 500, 1000, 1500, 2000 and 2500 μm distal to the lesion site in 8 longitudinal sections through each nerve (n = 6 rats/12 optic nerves/treatment). Optic nerve diameter was recorded using Axiovision Software (Zeiss) and the total number of axons per nerve extending distance *d* (Σ*a*_*d*_), in an optic nerve of radius *r* calculated by summing over all sections with a thickness (*t*) of 15 μm according to the following formula:

$\sum a_{d}=\pi r^{2}\times \left(\text{average}\;\;\text{axons}\;{\;\mathrm{mm}}^{-1}\right)/t.$

### 2.14 Protein extraction and western blotting

Protein extraction and western blots were performed according to our previously published methods (Vigneswara et al., 2013, 2014). Briefly, tissues from 6 rats/treatment (*i.e.* 12 retinae) were homogenised in ice-cold lysis buffer (20 mM Tris, pH 7.4, 2 mM EDTA, 0.5 mM EGTA, 150 mM NaCl, 1% NP-40 and 5 μl/ml protease inhibitor cocktail) and 40 μg of total protein was resolved in 12% SDS gels, transferred to polyvinylidene fluoride membranes (Millipore, Watford, UK) and probed overnight at 4 °C with appropriate antibodies. Bands were detected with HRP-labelled secondary antibodies (GE Healthcare, Buckinghamshire, UK) using an enhanced chemiluminescence kit (GE Healthcare).

### 2.15 Densitometry

Western blots were scanned into Adobe Photoshop (Adobe Systems Inc., San Jose, CA, USA) keeping all scanning parameters the same between blots. Bands were then analysed using the built-in-macros for gel analysis in ImageJ (NIH, USA, http://imagej.nih.gov/ij) and means ± SEM plotted in Microsoft Excel (Microsoft Corporation, CA, USA) (Ahmed et al., 2005, 2006a, 2011; Douglas et al., 2009). The integrated density of each protein band in each lane was derived from 3 separate blots from 3 independent experiments.

### 2.16 Statistical analysis

Significant differences were calculated between sample means using GraphPad Prism (GraphPad Software Inc. Version 4.0, CA, San Diego, USA) by one-way analysis of variance (ANOVA) followed by *post-hoc* testing with Dunnett's method and P < 0.05 was accepted as significant.

## 3 Results

### 3.1 PEDF-34 promoted significantly more neuron survival and neurite outgrowth than PEDF-44

**Fig. 1:**
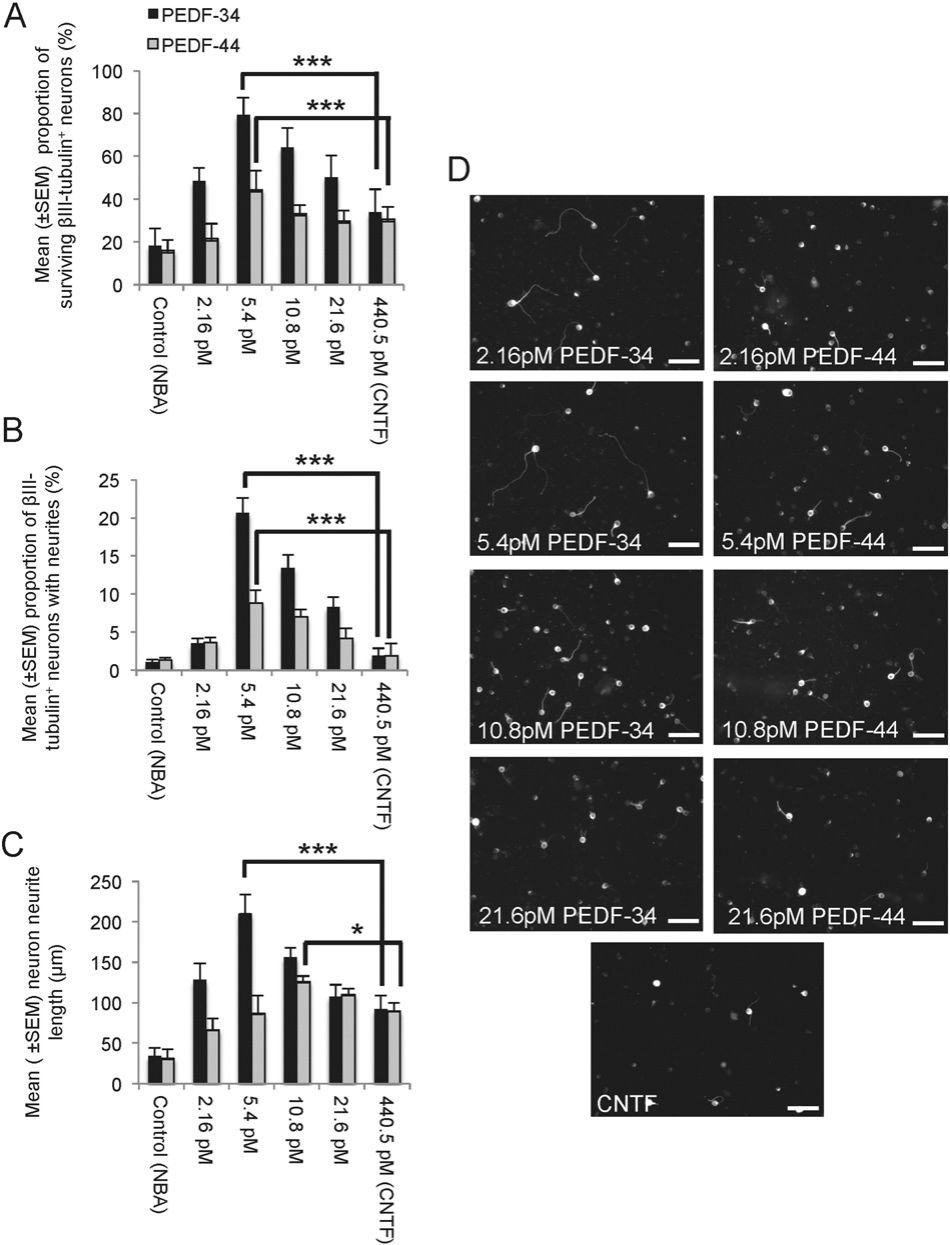
Retinal neuron survival and axon regeneration after exposure to PEDF-34 and PEDF-44. (A) Retinal neuron proportions after incubation with increasing concentrations of PEDF-34/PEDF-44 and CNTF demonstrate that 5.4 pM of PEDF-34 and PEDF-44 optimally promotes neuronal survival. (B) The mean proportion of neurons with neurites and (C) the mean neurite length also increased with increasing concentration of PEDF-34 and PEDF-44 with peak values at 5.4 pM and 10.8 pM, respectively. (D) Representative images of βIII-tubulin^+^ neurons to demonstrate neurite outgrowth. *** = P < 0.0001; * = P < 0.05. Scale bars = 50 μm. Initial βIII-tubulin^+^ retinal neuron plating density in wells 1–3 = 1350, wells 4–6 = 1337 and wells 7–9 = 1387 neurons.

Both PEDF-34 and PEDF-44 promoted a dose-dependent increase in βIII-tubulin^+^ neuron survival and neurite outgrowth (Fig. 1A–D), probably through both direct effects on retinal neurons and indirect effects on other retinal cells. For example, the proportion of surviving βIII-tubulin^+^ neurons in control untreated cultures was 18 ± 8%, whilst the proportion of surviving βIII-tubulin^+^ neurons at the optimal concentration (5.4 pM) of PEDF-34 and PEDF-44 in cultures was 81 ± 8 and 44 ± 9%, respectively (Fig. 1A). CNTF (positive control) promoted the survival of 30–34 ± 6% βIII-tubulin^+^ neurons after 4 days in cultures. These results show that PEDF-34 treatment resulted in a 4.5-fold increase in βIII-tubulin^+^ neuron numbers, whilst survival in CNTF or PEDF-44-treated cultures was 1.9- and 2.4-fold increased, respectively, compared to untreated cultures.

The mean proportion of βIII-tubulin^+^ neurons with neurites and the mean neurite length was optimal at 5.4 pM in PEDF-34 treated cultures, reaching a maximum of 21 ± 2% βIII-tubulin^+^ neurons with neurites (Fig. 1B) and a mean neurite length of 211 ± 23 μm (Fig. 1C and D). With PEDF-44, the proportion of βIII-tubulin^+^ neurons with neurites was 9 ± 1%, a response that peaked at 5.4 pM (Fig. 1B), whilst mean neurite length peaked at 126 ± 7 μm at 10.8 pM (Fig. 1C and D). PEDF-34 promoted 1.7-, 2.2- and 6.3-fold longer neurites and 2.3-, 10.5-, and 21-fold more βIII-tubulin^+^ neurons with neurites than in PEDF-44, CNTF or untreated cultures. These results demonstrate that PEDF-34 is significantly more neuroprotective and neuritogenic than PEDF-44, an effect that has not been previously described.

### 3.2 Intravitreal injection and eye drops of PEDF-34 promoted RGC survival *in vivo*

**Fig. 2:**
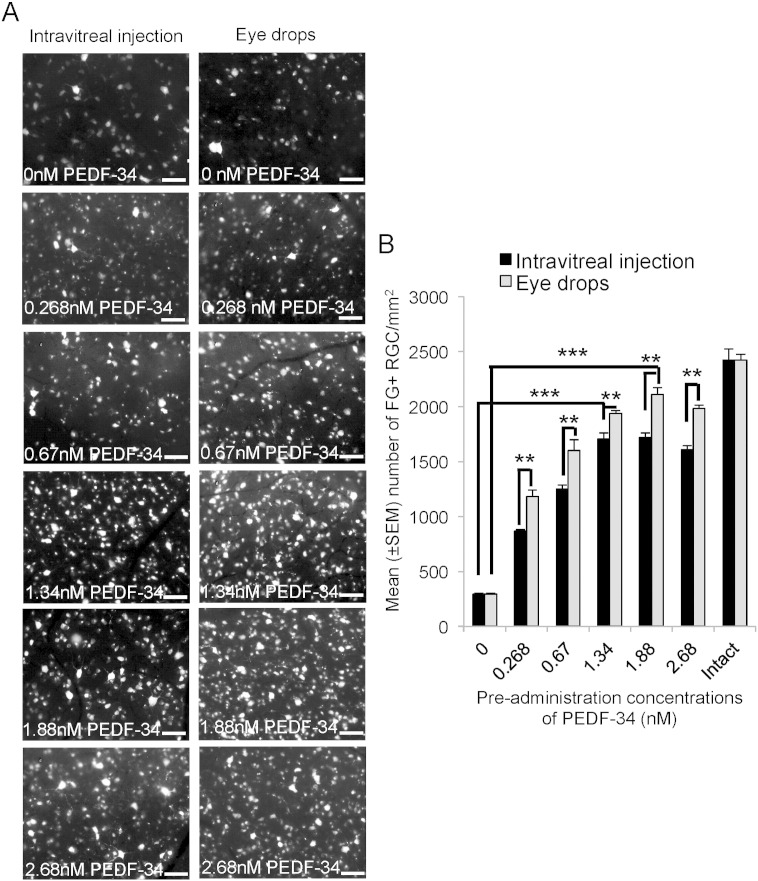
Weekly intravitreal injection of PEDF-34- and daily eye drop-mediated RGC neuroprotection is dose-dependent. (A) Representative images to show FG^+^ RGCs at 21 days after ONC and weekly intravitreal injections of 0, 0.268, 0.67, 1.34, 1.88 and 2.68 nM PEDF-34. (B) The number of FG^+^ RGCs present after delivery of different doses of PEDF-34 and in intact controls. Scale bars = 50 μm. ** = P < 0.001, *** = P < 0.0001.

We have already shown that intravitreal delivery, using the same dosing regime (0, 7 and 14 days after ONC) and equimolar concentrations of full length PEDF, promoted the survival of 55% of RGCs from death at 21 days after ONC, whilst administration of CNTF after ONC promoted limited RGC axon regeneration (Vigneswara et al., 2013). Here we wished to confirm that, *in vivo*, PEDF-34 was more neuroprotective than full length PEDF. After intravitreal injection of vehicle + 0 nM PEDF-34, 295 ± 15 FG^+^ RGC/mm^2^ survived 21 days after ONC + PBS (0 nM PEDF-34) (Fig. 2A and B). Increasing the dose of PEDF-34 caused a dose-dependent increase in the number of FG^+^ RGC surviving, achieving a maximum of 1720 ± 38 RGCs/mm^2^ using 1.88 nM. RGC survival did not improve by increasing the dose of PEDF-34 to as much as 2.68 nM (Fig. 2A and B). These results show that an intravitreal injection of an optimal dose of 1.88 nM PEDF-34 promoted 71% survival compared RGC numbers in intact controls.

 We also investigated in the same experiment, if daily eye drops of PEDF-34 were effective in enhancing RGC survival at 21 days after ONC. The number of FG^+^ RGCs counted in the control ONC + PBS-treated group was 292 ± 11 cells/mm^2^ (Fig. 2A and B). Increasing the concentration of PEDF-34 in the eye drops increased the number of FG^+^ RGCs; a response that peaked at a concentration of 1.88 nM when the number of surviving FG^+^ RGCs increased to 2110 ± 24 cells/mm^2^ (Fig. 2A and B), representing RGC survival of 87% compared to intact controls. These results suggest that PEDF-34 eye drops were more effective at protecting RGCs from death after ONC than intravitreal injections.

### 3.3 PEDF-34 accumulated in the eye after delivery by eye drops

**Fig. 3:**
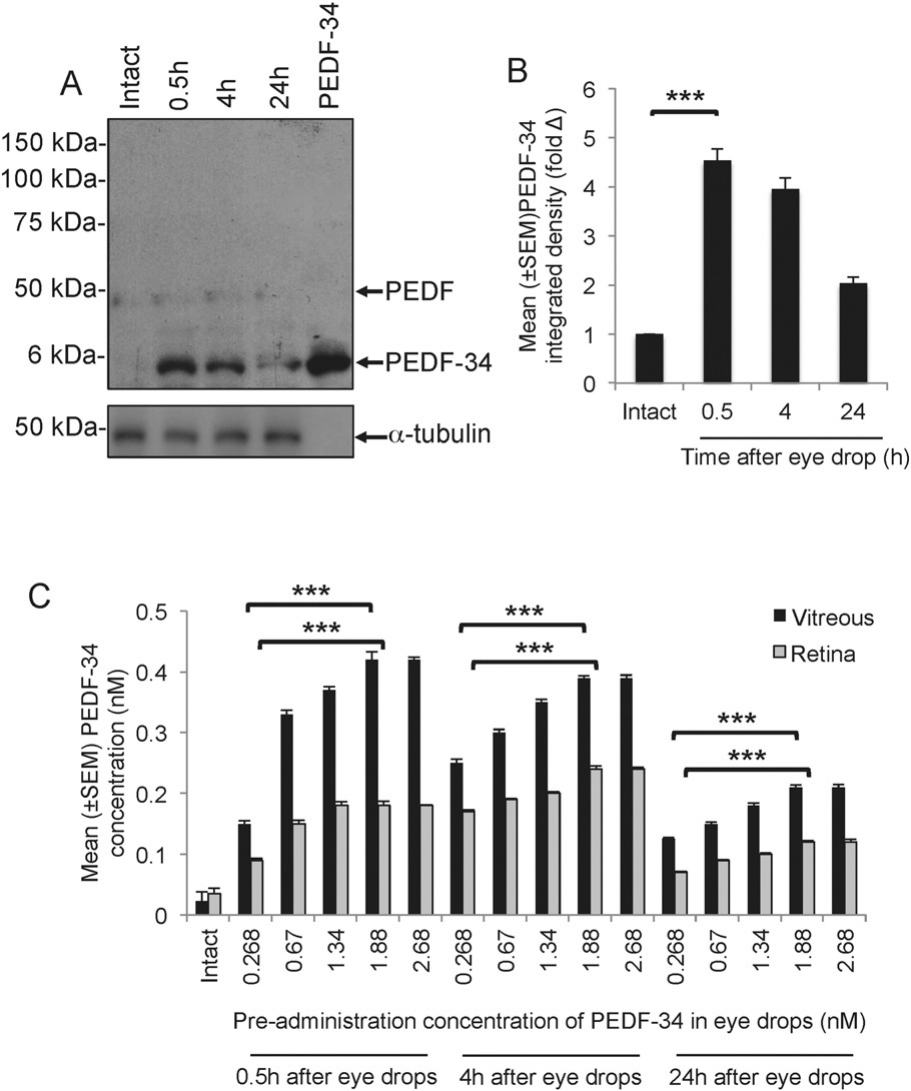
PEDF-34 detection in the vitreous and the retina after topical application of an optimal 1.88 nM neuroprotective dose. (A) Representative full-length western blot showing no changes in endogenous full length PEDF after a single delivery of PEDF-34 by eye drops whilst PEDF-34 levels increased within 30 min then slowly reduced over the next 24 h. Also included in a positive control lane, which contained 10 μg of PEDF-34. (B) Densitometry shows that PEDF-34 levels were 4.9-, 3.8- and 1.8-fold higher than the endogenous low levels of PEDF-34 seen in intact eyes, at 0.5, 4 and 24 h after a single administration of PEDF-34 eye drops. (C) Concentrations of PEDF detected by ELISA in the vitreous and the retina demonstrating a dose-dependent increase in total PEDF at 0.5, 4 and 24 h after a single occasion eye drop delivery.

Small fragments of PEDF in vehicle, topically applied to the surface of the eye enter into the vitreal chamber and access the retina (Liu et al., 2012). We wished to establish if PEDF-34 accumulated in the posterior chamber after delivery to the corneal/conjunctival surface by eye drops. No changes in endogenous full-length PEDF were detected in the vitreous using western blot and subsequent densitometry detected a 4.9-, 3.8- and 1.8-fold increase in PEDF-34 in both vitreous and retina after delivery of the optimal 1.88 nM concentration of PEDF-34 by eye drops after 0.5 h, 4 h and 24 h, respectively (Fig. 3A and B). However, little or no changes in endogenous full-length PEDF were observed, and little or no PEDF-34 was detected in intact eyes (Fig. 3A). A positive control lane (10 μg of PEDF-34 peptide; Fig. 3A) indicated that the antibody detected only changes in PEDF-34 and no other potential breakdown products of PEDF were detected. These results demonstrated that after eye drop delivery of PEDF-34: (1), the levels of endogenous full length PEDF are barely affected; (2), the polyclonal antibody to full length PEDF predominantly recognises PEDF-34; and (3), that PEDF-34 accumulates in the vitreous within 0.5 h and attain concentrations twice that of untreated eyes after 24 h.

We also performed ELISA with our polyclonal antibody to full length PEDF to detect the levels of total PEDF in the vitreous and retina at 0.5, 4 and 24 h after application of a single eye drop dose. Since endogenous full-length PEDF levels do not change, any measured changes must correspond to the accumulation of exogenous PEDF-34. ELISA in the retina detected high levels of PEDF-34 and vitreous within 0.5 h, declining slightly after 4 h with a further decline at 24 h (Fig. 3C). For example, increasing the concentrations of PEDF-34 in eye drops led to increased levels of total PEDF in the vitreous and retina at 0.5 h, peaking with a dose of 1.88 nM at 0.42 ± 0.01 nM and 0.18 ± 0.007 nM, respectively (Fig. 3C). At 4 h, peak levels of total PEDF in the vitreous and retina were 0.39 ± 0.004 nM and 0.24 ± 0.005 nM, whilst, at 24 h, peak levels in the vitreous and retina were 0.21 ± 0.004 nM and 0.12 ± 0.002 nM, respectively (Fig. 3C). These data confirm that high concentrations of PEDF-34 accumulate in the posterior chamber of the eye after topical eye drop application.

**Fig. 4:**
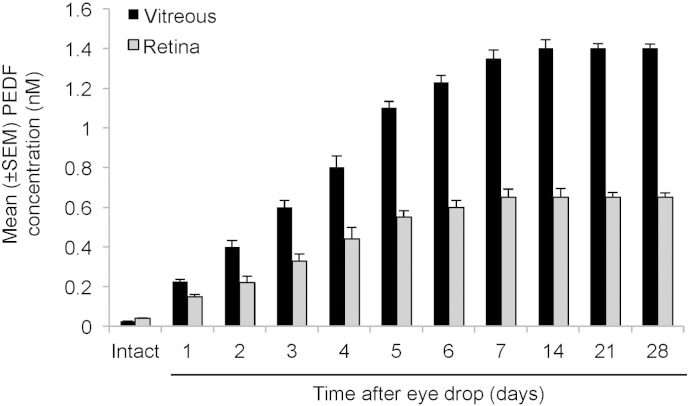
PEDF-34 levels in the vitreous and retina monitored by ELISA over 28 days after daily eye drop application of the optimal 1.88 nM neuroprotective dose. The levels of total PEDF (full length and fragments) in the vitreous and retina increased over time to a peak at 14 days, after which a plateau in concentration was reached despite continued eye drop application.

We next investigated whether eye drops prepared with the optimal concentration of PEDF-34 of 1.88 nM, when applied daily, could maintain high levels of PEDF-34 in the vitreous and retina for 28 days. Eyes receiving eye drops were carefully monitored with an ophthalmoscope for signs of corneal reactions to PEDF-34, including redness, inflammation or opacity throughout the duration of the experiment. None of the PEDF-34 treated eyes exhibited any of these symptoms. Daily PEDF-34 eye drops induced a gradual increase in the levels of PEDF-34 in both the vitreous and the retina over the first 7 days which peaked at 14 days to 1.4 ± 0.045 nM and 0.65 ± 0.025 nM, respectively (Fig. 4). After extending the treatments for 28 days, no further increase in the level of PEDF-34 was apparent in either the vitreous or retina (Fig. 4). These results suggest that levels of PEDF-34 gradually increase in the vitreous and retina over the first 14 days to a plateau level, which is maintained thereafter with continued eye drops.

### 3.4 Daily eye drops of PEDF-34 promoted more RGC axon regeneration than weekly intravitreal injections of PEDF-34

**Fig. 5:**
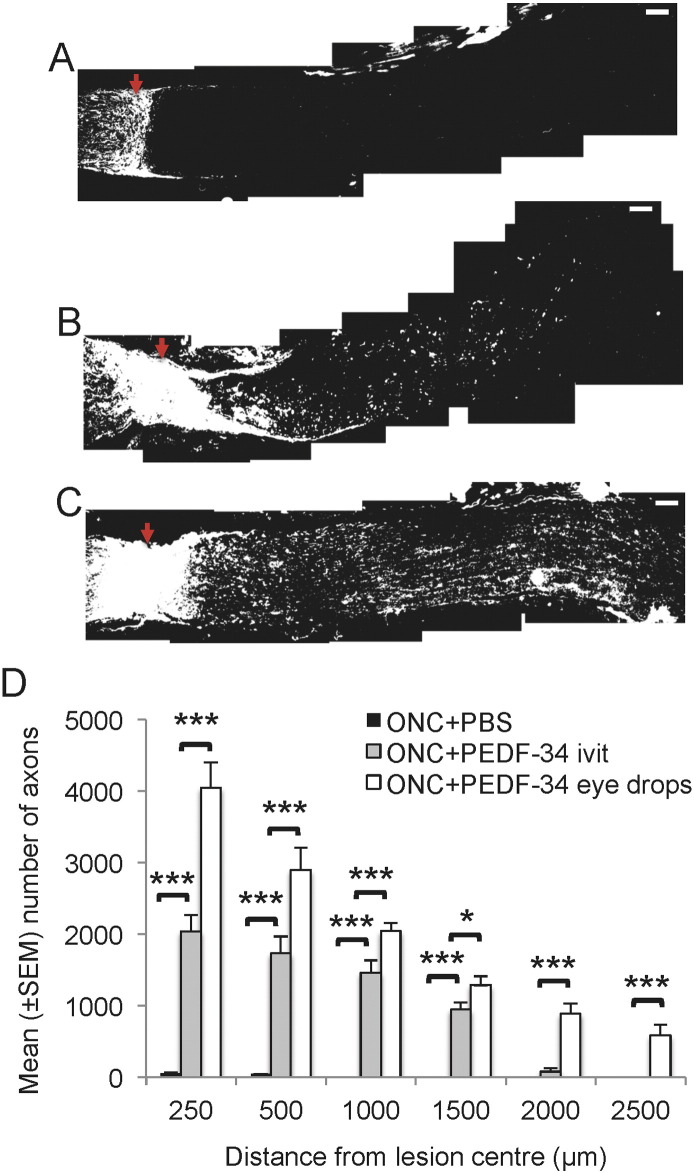
Weekly intravitreal injections and daily eye drop application of PEDF-34 promote RGC axon regeneration at 21 days after ONC. Representative GAP-43^+^ immunohistochemistry to show axons growing in the optic nerve after (A), ONC + PBS, (B), ONC + weekly intravitreal injections of PEDF-34 and (C), ONC + daily eye drops of PEDF-34 (scale bars = 100 μm). (D) Quantification of the number of GAP-43^+^ axons growing in the distal optic nerve stump at 250, 500, 1000, 1500, 2000 and 2500 μm from the lesion centre. * = P < 0.05, *** = P < 0.0001, ivit = intravitreal. Red arrow = lesion epicentre.

Few GAP-43^+^ axons were observed at 21 days after ONC in the proximal optic nerve stump of control eyes treated with PBS eye drops and only 56 ± 11 and 44 ± 4 were present at 250 and 500 μm, respectively, beyond the lesion site and in the distal stump (Fig. 5A and D). After weekly intravitreal delivery of the optimal 1.88 nM concentration of PEDF-34, numerous RGC axons were seen in the proximal optic nerve stump, whilst 2038 ± 234, 1733 ± 237, 1462 ± 175, 945 ± 100 and 79 ± 44 axons grew up to 200, 500, 1000, 1500 and 2000 μm from the lesion site, respectively, into the distal optic nerve stump (Fig. 5B and D). However, the greatest number of RGC axons was observed in the optic nerve of the PEDF-34 eye drop-treated groups when 4049 ± 355, 2893 ± 315, 2043 ± 120, 1285 ± 126, 884 ± 139, 582 ± 155 axons were present at 200, 500, 1000, 1500, 2000, and 2500 μm distal from the lesion site, respectively (Fig. 5C and D). These results demonstrated that over a period of 21 days, daily PEDF-34 eye drops were more axogenic (P < 0.0001) than weekly intravitreal injections of equimolar concentrations of PEDF-34.

## 4 Discussion

In the present study, we demonstrate that PEDF-34 is both neuroprotective and axogenic *in vitro* and *in vivo* for adult rat retinal neurons/RGCs. PEDF-34 is also more effective in promoting RGC survival and axon regeneration compared to full-length PEDF or PEDF-44. Delivery of PEDF-34 by eye drops protected 87% of RGCs from death after ONC, whilst weekly intravitreal injection of PEDF-34 supported 71% of RGCs from death. Daily PEDF-34 eye drops also promoted the regeneration of more RGC axons than did weekly intravitreal injections. In addition, daily eye drop delivery of PEDF-34 is detected in the vitreous within 0.5 h and accumulates in there and in the retina in high concentrations by 5 h and reaches a plateau of concentration at 14 days, when further eye drop applications maintain but do not increase levels of PEDF-34. Thus, daily eye drops of PEDF-34 are more RGC neuroprotective and axogenic compared to weekly intravitreal PEDF-34 injections.

Our results with PEDF-34 on retinal neuron/RGC protection do not substantiate those of Bilak et al. (2002), a condition that may be explained by differences in the neuronal phenotypes used, *i.e.* embryonic day 16 motor neurons used by Bilak et al. (2002), whilst we cultured adult sensory neurons in this current study. It is also well recognised that embryonic neurons behave differently to adult neurons and one significant factor in our observed effects of PEDF-34 on adult retinal neuron/RGC neuroprotection and axon regeneration might be that cyclic adenosine monophosphate (cAMP) levels are attenuated in adult neurons whilst embryonic neurons require neurotrophic factors for their survival. The same PEDF-34 sequence was also reported by others to be non-neurotrophic and anti-angiogenic by (Alberdi et al., 1999; Filleur et al., 2005). However, our current study shows that PEDF-34 is highly neuroprotective and axogenic to adult rat retinal neuron/RGC both *in vitro* and *in vivo* and is also more potent than the currently accepted neurotrophic fragment of PEDF, PEDF-44 (Bilak et al., 2002; Filleur et al., 2005). Moreover, we do not yet know why PEDF-34 was better than PEDF-44 in terms of neuroprotection and RGC neurite outgrowth but this is currently under investigation. PEDF-34 and PEDF-44 appeared to be equally stable in culture media and retained their neurotrophic properties after long-term storage. It is also not known whether PEDF-34 and PEDF-44 bind different receptors, however further work in this area would help clarify these issues.

The mechanism of enhanced retinal neuron/RGC survival after PEDF-34 treatment is unknown, although exogenous PEDF-34 may down regulate the apoptotic genes caspase-2, calpain and mitogen activated protein kinase (MAPK)-1 after binding to one or more the high affinity PEDF receptors, stimulating downstream phospholipase A2 enzymatic activity (Tombran-Tink and Barnstable, 2003). Inhibition of both nuclear kappa-light-chain-enhancer of activated B cell (NFκB) and extracellular signal-related kinase (ERK)-1/2 pathways abrogates the protective effects of PEDF in cultured retinal neurons, suggesting that the NFκB and ERK1/2 pathways activated by PEDF may mediate retinal neuron/RGC neuroprotection (Pang et al., 2007; Tombran-Tink, 2005; Tombran-Tink and Barnstable, 2003). Activation of the NFκB pathway induces the expression of brain-derived neurotrophic factor (BDNF), nerve growth factor (NGF), B-cell lymphoma-extra large and superoxide dismutase (Courtois, 2005; Wu and Kral, 2005), whilst PEDF activated ERK1/2 modulates kinases, phosphatases, transcription factors and regulators of apoptosis (Rubinfeld and Seger, 2005; Yoon and Seger, 2006), which all enhance retinal neuron/RGC survival. Enhanced retinal neuron/RGC survival may also be facilitated indirectly by retinal glia and other neurons since our retinal cultures contained mixed retinal cells. However, we have previously characterised our cultures and shown that activated glia, which secrete additional neurotrophic factors, were absent in our retinal cultures (Suggate et al., 2009) and therefore we expect the contribution of indirect effects on retinal glia and other neurons to be minimal as opposed to the direct effects of PEDF-34 on retinal neurons/RGCs.

Both extrinsic and intrinsic factors regulate axon sprouting and elongation supported by the observation that PEDF is included among the neurotrophic factors that activate the ras–raf–MAPK and the phosphoinositide 3-kinase (PI3K)–protein kinase B (Akt) signalling pathways which are both axogenic (Koeberle and Ball, 2002). Five-fold more retinal neuron/RGC neuritogenesis/axogenesis was stimulated by PEDF-34 when compared to untreated controls, confirming that PEDF-34 is a potent axogenic factor. Inhibiting apoptosis after either Bax knockout or overexpression of Bcl-2, whilst suppressing either MAPK or PI3K, partially arrests axon regeneration, whilst inhibiting both together, completely suppresses axon elongation (Mo et al., 2002). Conversely, stimulating either the PI3K or Akt pathways increases axon thickness and branching whilst blocking raf, PI3K and Akt suppresses cAMP potentiated axon outgrowth (Mo et al., 2002). Although we showed that secreted retinal glia-derived PEDF may supplement endogenous titres of PEDF (Vigneswara et al., 2012), exogenous PEDF-34 delivery does not affect endogenous levels of full length PEDF and thus retinal glia-derived PEDF does not contribute to the enhanced retinal neuron/RGC neuroprotection and axogenesis observed in our current study. Although PEDF is constitutively expressed by pigment, ciliary and corneal epithelial cells and also by Müller glia, retinal astrocytes and neurons/RGC, the levels of PEDF are generally low. Furthermore, we detected a weak band correlating to PEDF-34 in intact retina, suggesting that PEDF-34 may naturally be present in low amounts but this has never been reported by others and requires further investigation. Therefore, exogenous PEDF-34 delivery, especially through the use of eye drops is potentially a non-invasive, therapeutically advantageous strategy to promote RGC survival and axon regeneration. It is widely accepted that different signalling pathways regulate neuron survival and axon regeneration since both responses can be isolated. For example, we have shown previously that intravitreal and intra-optic nerve inflammation is retinal neuron/RGC protective but only the former promotes axon regeneration (Ahmed et al., 2010). Therefore, one particular advantage of PEDF-34 is that both retinal neuron/RGC survival and axon regeneration are significantly promoted, implying that multiple axon growth and survival pathways are activated by PEDF-34.

Other commonly used retinal neuron/RGC neuroprotective factors include BDNF, glial-derived neurotrophic factor (GDNF), neurotrophin (NT)-3, NT-4/5, as well as caspase inhibitors (Cheng et al., 2002; Kermer et al., 1998; Kurokawa et al., 1999; Monnier et al., 2011; Parrilla-Reverter et al., 2009). Although intravitreal injections of BDNF protect 100% of RGC for 7 days, efficiency declines to 60% by 21 days. Most neuroprotective strategies tested support the survival of a maximum of 60% of RGC at 2 weeks after injury (Cheng et al., 2002; Kermer et al., 1998; Kurokawa et al., 1999; Monnier et al., 2011; Parrilla-Reverter et al., 2009) whilst combinations of agents such as GDNF + BDNF and BDNF + Tropomysin receptor kinase (Trk)B support up to 80% RGC survival, but very few of these combinations of neurotrophic factors support significantly greater axon regeneration than that obtained with CNTF. PEDF promotes more survival of retinal neuron/RGC *in vitro* and *in vivo* than any other neurotrophic factor thus far reported, and is also one of the most retinal neuron/RGC axogenic factors.

Drug administration to the retina and vitreous body is challenging. Drugs applied to the surface of the eye rarely penetrate the ocular surface or enter into the vitreous body and the retina (Gaudana et al., 2010). Biological barriers such as the cornea, conjunctiva and the tear film limit penetration and bioavailability of topical drug preparations to the posterior segment of the eye and therefore direct injection of drugs to the vitreous is commonly used to target drugs to the retina (Ayalasomayajula and Kompella, 2005; Carneiro et al., 2009; Eldaly and Styles, 2009; Yasukawa et al., 2001) but has the risk of significant complications including cataracts, vitreous haemorrhages and retinal detachment. There are several colloidal drug delivery systems such as liposomes (Hathout et al., 2007; Kaur et al., 2004; Law et al., 2000), niosomes (Abdelbary and El-Gendy, 2008), nanoparticles (Agnihotri and Vavia, 2009) and nanoemulsions (Badawi et al., 2008) that enhance bioavailability of drugs to the retina after eye drop administration. For example, modified liposomes made from poly-l-lysine, are cationic and effectively deliver peptides to the back of the eye (Makhlof et al., 2011; Murata et al., 2012), but whether therapeutic proteins or peptides reach the posterior segment of the eye is controversial. However, after delivery of insulin (5.8 kDa) by eye drops combined with a penetration enhancer, insulin is localised to the Lewis rat retina and accumulates in the optic nerve (Koevary et al., 2002). In adult rats, high levels of ^125^I-labelled nerve growth factor (26 kDa) accumulates in the retina and optic nerve peaking at 6 h after topical administration (Lambiase et al., 2005).

Antibody fragments (28 kDa) also penetrate into the rabbit vitreous (Williams et al., 2005), whilst ESBA105, an anti-tumour necrosis factor single-chain antibody (26 kDa) reaches the retina after topical administration in New Zealand rabbit eyes without the use of penetration enhancers (Furrer et al., 2009). In this latter study, topical delivery of ESBA105 was advantageous over intra-vein injections achieving 25-fold higher drug levels, suggesting that an anterior trans-scleral passage of the drug may have occurred without trans-corneal passage (Furrer et al., 2009). Our result with 3.7 kDa PEDF-34 is entirely consistent with the above reports and suggests that PEDF-34 passes into the posterior chamber without the need for drug carriers and accumulates in the vitreous quickly and efficiently (within 30 min), achieving high retinal concentrations by 4 h. The diffusion of PEDF-34 is partly due to the small size of this sequence, it's hydrophilic nature and it's daily administration. PEDF-34 would therefore diffuse through the cornea and the subconjunctiva and probably through diffusion into ocular surface vasculature, all of which will facilitate the transport of PEDF-34 into the retina. Indeed, a 2.1 kDa fragment, labelled with fluorescein was localised throughout the cornea and reached therapeutic levels into the retina after 4 h (Liu et al., 2012).

As indicated, topically applied PEDF-34 peptide may reach the posterior chamber through multiple routes, including diffusion through the cornea and conjunctiva since the cornea and conjunctiva were intensely positive for fluorescein-labelled PEDF (Liu et al., 2012). PEDF-34 may also diffuse into ocular surface blood vessels, thereby passing into the RPE-choroid and inner retina vasculature (Liu et al., 2012). As for why PEDF-34 eye drops should be better than intravitreal injections of PEDF-34 remains to be investigated. We suspect that this may be due to the fact that intravitreal injection delivers a bolus of PEDF-34 that may have beneficial neuroprotective and axogenic properties in the eye prior to its clearance before the next injection. However, since eye drops are administered daily, this sustains maximal neuroprotective and axogenic properties throughout the time of its administration. Eye drops of PEDF-34 therefore counter the natural ONC injury-related deleterious effects observed.

In conclusion, contrary to early reports that PEDF-34 is not neuroprotective, we demonstrate that PEDF-34 is more retinal neuron/RGC neuroprotective and axogenic than either full-length PEDF or PEDF-44. Moreover, the potent retinal neuron/RGC protective and axogenic effects of eye drops comprising PEDF-34 have potential, non-invasive convenient and more effective use than intravitreal injections in the treatment of neuropathies and diseases of the eye featuring retinal neuron/RGC death, such as glaucoma.

## Acknowledgements/disclosures

This work was funded by the Wellcome Trust Grant no. 092539/Z/10/Z (ZA).

All authors declare no financial disclosures.

## Figures and Tables

**Table 1:** List of antibodies, their source and dilutions used in this study.

Antibody	Host	Source	Use	Dilution
*Primary*				
βIII-Tubulin	Mouse	Sigma, Poole, UK	IHC	1:200
PEDF	Rabbit	R&D Systems, Oxford, UK	IHC/WB	1:400/1:500
GAP43	Mouse	Invitrogen, Paisley, UK	IHC	1:400
α-Tubulin	Mouse	Abcam, Cambridge, UK	WB	1:1000

*Secondary*				
Alexa 488	Goat	Invitrogen, Paisley, UK	IHC	1:400
Texas Red	Donkey	Invitrogen, Paisley, UK	IHC	1:400
